# RETRACTION

**DOI:** 10.1111/jocd.15936

**Published:** 2023-07-26

Cavallini M, De Luca C, Prussia G, Raichi M. PN‐HPT® (Polynucleotides Highly Purified Technology) in facial middle third rejuvenation. Exploring the potential. *J Cosmet Dermatol*. 2022; 21: 615‐624. https://onlinelibrary.wiley.com/doi/full/10.1111/jocd.14578. The above article, published online on November 17, 2021, in Wiley Online Library (wileyonlinelibrary.com), has been retracted by agreement between the journal's Editor‐in‐Chief, Michael H Gold, and Wiley Periodicals, LLC. After publication, concerns were raised by a third party regarding possible duplication between the multipaneled images within Figures 3 and 4—specifically, that they did not show the patients before and after treatment as reported. As part of the journal's investigation, the authors provided the raw, unmodified images for review. The analysis of the raw images resolved the issues for Figure 3. However, concerns remained about the duplication of images between the reported baseline and posttreatment images in Figure 4 that the authors were unable to provide a satisfactory explanation for. As a result, the conclusions reported in the article are no longer considered reliable.

